# A novel strategy for detecting multiple mediators in high-dimensional mediation models

**DOI:** 10.3389/fpsyt.2025.1611761

**Published:** 2025-12-15

**Authors:** Pei-Shan Yen, Zhaoliang Zhou, Soumya Sahu, Debarghya Nandi, Olusola Ajilore, Dulal Bhaumik

**Affiliations:** 1 Division of Epidemiology and Biostatistics, University of Illinois at Chicago, Chicago, IL, United States; 2 Department of Psychiatry, University of Illinois at Chicago, Chicago, IL, United States

**Keywords:** biomarker detection, mediation analysis, overestimation, choice of penalty, LASSO, Pathway LASSO

## Abstract

This article presents a novel methodology for detecting multiple biomarkers in high-dimensional mediation models by utilizing a modified Least Absolute Shrinkage and Selection Operator (LASSO) alongside Pathway LASSO. This approach effectively addresses the problem of overestimating direct effects, which can result in the inaccurate identification of mediators with nonzero indirect effects. To mitigate this overestimation and improve the true positive rate for detecting mediators, two constraints on the *L*_1_-norm penalty are introduced. The effectiveness of the proposed methodology is demonstrated via extensive simulations across various scenarios and compared against other popular mediation methods, highlighting its robustness and reliability under different conditions. Furthermore, a procedure for selecting an optimal threshold for dimension reduction using sure independence screening is introduced, enhancing the accuracy of true biomarker detection and yielding a final model that is both robust and well-suited for real-world applications. To illustrate the practical utility of this methodology, the results are applied to a study dataset involving patients with internalizing psychopathology and another dataset involving patients with late-life depression, showcasing its applicability in clinical settings. Overall, this methodology signifies a substantial advancement in biomarker detection within high-dimensional mediation models, offering promising implications for both research and clinical practices.

## 1 Introduction

Understanding the impact of pharmacological interventions on neuroimaging biomarkers is crucial for improving therapeutic outcomes in brain disorders. Identifying neuroimaging biomarkers—such as brain connectivity, structural changes, metabolic activity, and functional responses—offers valuable insights into the intricate interactions within neural networks. Among these biomarkers, brain connectivity is particularly significant, as it reflects the interactions between distinct brain regions within both cortical and subcortical networks. Aberrant changes in brain connectivity can lead to neurological disorders, resulting in dysfunctions in cognitive processes that manifest as abnormal physical and mental behaviors. By assessing these relationships, researchers can develop more effective treatments, facilitate early diagnosis, and tailor interventions, such as neuromodulation for individuals with neurological disorders.

Brain connectivity encompasses effective connectivity (EC), structural connectivity (SC), and functional connectivity (FC). FC, in particular, reflects the temporal correlation of neurophysiological events across spatially separated neural assemblies, providing critical insights into the coordination and integration of brain activity during various cognitive processes (1–4). Our research aims to elucidate the neural mechanisms of FC that connect therapeutic interventions for improvements in neurobehavioral outcomes. To achieve this, we develop a high-dimensional mediation model that delineates neural processes, with FC measures acting as mediating variables. This approach emphasizes the importance of decomposing treatment effects into distinct pathways, thereby offering a more comprehensive understanding of the underlying neurobiological mechanisms.

High-dimensional mediation models encounter significant challenges in identifying neuroimaging biomarkers, primarily due to issues related to small sample sizes, which lead to unstable parameter estimates and insufficient statistical power (5). To mitigate these challenges, various dimension reduction strategies have been extensively investigated. In our pursuit of improved model interpretability and therapeutic efficacy, we focus on regularization methods. A spectrum of regularization methods including minimax concave penalty (MCP) (6, 7), Least Absolute Shrinkage and Selection Operator (LASSO) (8) and its extensions, such as de-biased LASSO (DBL) (9), adaptive LASSO (AdL) (10), and elastic net (EN) (11), explicitly penalize the total indirect effect (TIE). Whereas Pathway LASSO (12–14), built upon EN framework effectively identifies mediators along its pathway, thereby enhancing the robustness of the analysis.

Implementing LASSO or Pathway LASSO in mediation models poses several challenges related to parameter estimation. A primary issue is the overestimation of the direct effect (DE) (15). This problem intensifies with an increasing number of mediators (*p*) or a decreasing sample size (*n*). The overestimation arises because when TIE is not fully accounted for during the estimation process, it contributes to an erroneous estimation of DE, as the total effect (TE) is the sum of DE and TIE. For instance, LASSO focuses on estimating the parameters associated with TIE. Without any explicit constraints on DE, it tends to overestimate DE and underestimate TIE, adversely impacting the model accuracy, especially the true positive rate (TPR) of mediators with nonzero effects. Even with Pathway LASSO, which implements an *L*_1_-norm penalty to DE, the problem persists.

The second challenge resides in the difficulty of accurately identifying mediators with nonzero effects, commonly known as true signals, which is essential for understanding the underlying mechanisms at play. Commonly used regularization methods, such as LASSO and Pathway LASSO, apply an *L*_1_-norm penalty with equal weights to both parameters involved in indirect effect (IE). However, the constraint of equal weights places excessive restrictions on one set of parameters, diminishing the likelihood of detecting small values of those parameters and potentially lowering TPR of IE. Therefore, exploring an alternative approach that employs a constraint with a reduced magnitude for the tuning parameter warrants further investigation.

The third challenge pertains to ultra-high-dimensional mediators, where the number of mediators significantly exceeds the sample size, leading to substantial computational difficulties and a marked reduction in statistical power. The application of regularization techniques in such models often results in an extremely low TPR for IE due to excessive noise and sparsity issues. To address these challenges, screening procedures like sure independence screening (SIS) (16) are frequently employed prior to regularization. However, a systematic approach for determining the appropriate dimensionality in mediation models remains elusive. Traditionally, the reduced dimension is set as *d* = *k*[*n/*log(*n*)], with *k* = 1 for linear models. In mediation analysis, where IE involves two parameter vectors, higher values of *k* (such as 2 or 3) have been suggested to enhance IE detection (6, 7, 17). Therefore, a methodological approach is needed to optimize the scaling factor *k*, achieving a balance between computational efficiency and the accurate detection of true signals in ultra-high-dimensional scenarios. This optimization is essential for improving the overall performance of mediation analysis in complex, high-dimensional settings.

The final challenge lies in the limited availability of R packages designed for high-dimensional mediator analysis that utilize regularization methods. Most existing R packages primarily focus on transformation techniques, such as those implemented in hdmed, or on Bayesian modeling approaches. To the best of the authors’ knowledge, few R packages such as regmed, is related to regularization methods; however, it is confined to LASSO penalty. This gap in the statistical landscape highlights the urgent need for the development of R packages that can address the optimization problem using a wider array of penalty strategies. Such advancements would significantly enhance researchers’ ability to conduct high-dimensional mediation analyses more effectively and accurately, ultimately contributing to the growth and innovation in the field.

In response to the aforementioned challenges, this research work is proposing novel strategies for tuning parameters, optimizing SIS procedures, and developing new R packages for identifying mediators in high-dimensional settings. Our work contributes four notable innovations as follows:

We develop strategy for improving the accuracy of DE estimation in high-dimensional mediation models through a tuning parameter for the *L*_1_-norm penalty. It introduces an additional constraint to enhance the TPR of IE by adjusting the tuning parameter associated with mediation framework parameters. A guideline for selecting an appropriate scaling factor for dimension reduction in ultra-high dimensional mediators is also presented, emphasizing the balance between computational cost and informative predictor retention. This guideline is theoretically supported by the sure screening property (16), which suggests that SIS is more likely to capture true signals as the reduced dimension increases. Finally, three R packages are developed to optimize high-dimensional mediation models, two of which connecting R to the Intel oneAPI Math Kernel Library and facilitating large-scale matrix computations, significantly improving computational efficiency.

This article is structured to guide the reader through our innovative approaches to address the identification of neuroimaging biomarkers for patients with internalizing psychopathology (IP) or late-life depression (LLD). It begins with two motivating examples in Section 2, which set the stage for understanding the complexities involved. In Section 3, we compare conventional regularization methods with our novel techniques, providing additional justifications for our approach. Section 4 presents extensive simulation results that demonstrate the effectiveness of our proposed method against other regularization methods, while Section 5 illustrates its application within the context of the IP study and the LLD study. Finally, the concluding section summarizes the key findings and suggests potential directions for future research, ensuring a well-rounded discussion of the topic.

## 2 Motivational example

### 2.1 IP study

This work draws inspiration from a study conducted by the University of Illinois at Chicago (UIC), which explored the effects of various treatments on individuals experiencing IP. The IP spectrum includes major depressive disorder (MDD), generalized anxiety disorder (GAD), post-traumatic stress disorder (PTSD), and other related conditions. In this study, participants were divided into two groups: the untreated IP group and the healthy control group (HC). Individuals with IP were randomly assigned to receive 12 weeks of treatment, either through Selective Serotonin Reuptake Inhibitors (SSRIs) or Cognitive Behavioral Therapy (CBT). To assess the impact of these treatments, psychiatric evaluations were conducted both before and after the intervention, employing the Depression Anxiety Stress Scales (DASS) (18), a transdiagnostic scale assessing symptoms across various disorders, to quantify changes in neurobehavioral outcome.

The study employed FC measures to identify disrupted brain connectivity in individuals with IP compared to HC. FC, which reflects the dynamic interactions among various brain regions during different mental processes, was assessed using resting-state functional Magnetic Resonance Imaging (rs-fMRI) data. The brain network was divided into 105 regions of interest (ROIs) based on the CONN atlas (19), with detailed parcellation provided in the supporting information, resulting in a total of 5,460 distinct FC links. Additional information regarding participant demographics, selection criteria, data processing, and imaging acquisition can be found in previously published articles (20) related to this research.

Antidepressants provide rapid treatment effects over short durations, prompting our analysis to focus on identifying potential biomarkers for neuromodulation that could expedite recovery in patients undergoing CBT or those with severe symptoms of IP. Our study examines 28 participants who received antidepressants (the IP-SSRI group) and completed rs-fMRI scans both before and after treatment. This group is compared to 27 participants in the HC group. By developing a mediation model, we aim to uncover neuroimaging biomarkers, particularly FC links, that mediate the relationship between treatment effects and improvements in neurobehavioral outcome.

### 2.2 LLD study

The second motivational example illustrates LLD, a prevalent disorder in adults over 55 characterized by depressive symptoms, cognitive deficits, and somatic complaints. A cross-sectional study conducted at UIC (21) included 23 participants, comprising 10 with LLD and 13 HC. The objective was to distinguish brain patterns between LLD and HC groups based on whole-brain FC alterations. For network construction, 87 ROIs were parcellated by the Freesurfer Desikan atlas (22), yielding 3,741 unique FC links. Neurobehavioral outcome was assessed using the Hamilton Depression Rating Scale (HAMD) (23).

## 3 Method

The high-dimensional mediation model introduced in this study is pivotal for understanding how mediators influence the connection between treatment and outcome. By identifying mediators that demonstrate nonzero IE, this model facilitates the discovery of potential biomarkers. The framework for mediation is clearly defined, and an innovative optimization method is presented, which combines feature selection with penalized estimation to enhance the model’s effectiveness.

### 3.1 Model definition

We develop a mediation model utilizing the linear structural equation modeling technique. The model encompasses *n* subjects, each assumed to be independent and identically distributed. Within this framework, *X* denotes the treatment exposure, represented as a binary variable (e.g., with *X* = 0 for the HC group and *X* = 1 for the IP-SSRI group), while *Y* serves as the continuous outcome variable, reflecting changes in DASS scores by calculating the difference between post-treatment and pre-treatment scores. The mediator vector, represented as ***M*** = (*M*_1_*,M*_2_*,…,M_p_*), captures the changes in FC that occur following the treatment.

(1) $$\{\begin{array}{l} M_{i}=X\alpha_{i}+\epsilon_{i},\;i=1,2,\ldots ,p\\ Y=X\gamma +\sum_{i=1}^{p}M_{i}\beta_{i}+\zeta \end{array}\;.$$

Errors associated with each mediator, $\epsilon_{i}$, are assumed to follow a multivariate normal distribution with a mean of zero and a covariance matrix **Σ**. The error of the outcome model, *ζ*, is assumed to follow a normal distribution with a mean of zero and a variance of $\sigma^{2}$. Furthermore, the errors associated with the mediators and the outcome variable are treated as independent.

Our mediation model assumes a parallel design (24), forgoing the incorporation of potential sequential relationships among mediators. This simplification is necessitated by insufficient empirical evidence in the extant literature supporting specific temporal orderings. Moreover, as demonstrated by Zhao and Luo (13), the parallel mediation model can be seen as a simplified version of a sequential mediation model under certain conditions. As a result, the interpretation of individual mediation effects remains consistent, regardless of the underlying structure or sequential dependencies among mediators.

### 3.2 Mediation effect

The parameter vector ***α*** = (*α*_1_*, α*_2_*,…,α_p_*), of dimension 1×*p*, characterizes the relationship between the treatment exposure *X* and the mediator vector ***M***, encapsulating the treatment effect on the variations in the FC mediators. Additionally, ***β*** = (*β*_1_*, β*_2_*,…,β_p_*), configured as a *p*×1 vector, describes the relationship between the mediator ***M*** and the outcome variable *Y*, quantifying the influence of FC mediators on the improvement in neurobehavioral outcomes.

Our mediation model, grounded in the above framework for potential outcomes (25), facilitates the examination of multiple mediation pathways while maintaining a clear delineation between DE and IE. This framework decomposes TE on an outcome into path-specific effects (26, 27) operating through distinct mediators. TE comprises two primary components: DE and TIE. The DE of treatment exposure ***X*** on outcome ***Y***, without the influence of mediators, is quantified by the parameter *γ*. TIE, also termed the mediation effect, represents the influence of treatment on the outcome through mediators, is computed using the product-coefficient method as $\sum_{i=1}^{p}\alpha_{i}\times \beta_{i}$. Within this formulation, IE through the *i*th mediator, denoted as *IE_i_*, is expressed as *α_i_* × *β_i_*. This approach allows for the decomposition of TIE into individual path-specific effects, enabling a nuanced analysis of complex mediation structures and providing insights into the relative contributions of each mediator to TIE.

### 3.3 Estimation of parameters

This research aims to improve the detection of mediators with nonzero IE. We adopt a simpler approach to streamline the estimation process by assuming a unit variance for the error distribution rather than employing a more intricate covariance matrix. This simplification will not compromise the consistency of the least-square estimators, provided that all variables are standardized to a unit scale (28). Accordingly, the log-likelihood *l*(***α***, ***β****, γ*), is specified as Equation 2:

(2) $$l\left(\alpha ,\beta ,\gamma \right)=tr\{{\left(M-X\alpha \right)}^{t}\left(M-X\alpha \right)\}+{\left(Y-X\gamma -M\beta \right)}^{t}\left(Y-X\gamma -M\beta \right).$$

#### 3.3.1 Commonly used methods

Two traditional regularization methods, LASSO and Pathway LASSO, are utilized to estimate IE for the high-dimensional model. When applied to our model, LASSO can be viewed in terms of Equation 3:

(3) $$\underset{\alpha ,\beta ,\gamma }{\text{argmin}}\{\frac{1}{2}l\left(\alpha ,\beta ,\gamma \right)+P_{1}\left(\alpha \right)+P_{2}\left(\beta \right)\}.$$

The *L*_1_-norm penalty functions are defined as $P_{1}\left(\alpha \right)=\;\lambda_{1\alpha }\sum_{i=1}^{p}\left|\alpha_{i}\right|$ and $P_{2}\left(\beta \right)=\;\lambda_{1\beta }\;\sum_{i=1}^{p}\left|\beta_{i}\right|$. These functions impose penalties on the model parameters ***α*** and ***β*** to enhance the accuracy of parameter selection.

Pathway LASSO can be viewed in terms of Equation 4, which is aimed at stabilizing estimates and minimizing estimation bias:

(4) $$\underset{\alpha ,\beta ,\gamma }{\text{argmin}}\{\frac{1}{2}l\left(\alpha ,\beta ,\gamma \right)+P_{1}\left(\alpha ,\;\beta \right)+P_{2}\left(\alpha ,\beta \right)+P_{3}\left(\gamma \right)\}.$$

This method, drawing from the principles of EN, introduces a penalty $P_{1}\left(\alpha ,\beta \right)=\kappa \sum_{i=1}^{p}\left[\left|\alpha_{i}\beta_{i}\right|+\nu \left(\alpha_{i}^{2}+\beta_{i}^{2}\right)\right]$ for the identification of path-specific effect, and this penalty remains convex when ν is greater than or equal to 0.5. An additional $L_{1}$-norm penalty $P_{2}\left(\alpha ,\beta \right)=\omega \sum_{i=1}^{p}\left(\left|\alpha_{i}\left|+\right|\beta_{i}\right|\right)$ is included to further shrink the individual parameters $\alpha_{i}$ and $\beta_{i}$, thereby improving the selection accuracy. Moreover, to address the overestimation of DE ( γ), an $L_{1}$-norm penalty for $P_{3}\left(\gamma \right)=\lambda_{\gamma }\left|\gamma \right|$ is also proposed in this method.

#### 3.3.2 The proposed method

We propose two tuning parameter strategies that relax the $L_{1}$-norm constraints to address challenges in traditional methods. The first strategy introduces an $L_{1}$-norm penalty $\lambda_{\gamma }\left|\gamma \right|$ for DE and establishes a minimum threshold for $\lambda_{\gamma }$ (specified as $\lambda_{\gamma }\geq c$) to address the issue of overestimation. This adjustment is particularly effective in reducing DE overestimation by compressing its estimated value toward zero, leading to a lower bias and variance of the estimated IE.

The second strategy aims to further enhance the TPR of IE by critically evaluating parameter estimation in $IE_{i}$ ( $\alpha_{i}\beta_{i}$) and improving the detection of mediators with nonzero impacts. This is achieved by reducing the magnitude of the tuning parameter, $\lambda_{1\beta }$, which is linked to the $L_{1}$-norm penalty for the parameter vector β, under the condition that $\lambda_{1\alpha }>\lambda_{1\beta }$. The justification for this strategy is detailed subsequently, focusing on the influence of the tuning parameters $\lambda_{1\alpha }$ and $\lambda_{1\beta }$ on parameter estimation.

- The parameter vector ***α*** consistently exhibits high TPR due to the unique structure of the mediation model. Each *α_i_* is independently estimated in its mediator model, see Equation 1, making the detection nonzero values of *α_i_* stable and less affected by the magnitude of the penalty $\lambda_{1\alpha }$, even for *α_i_* values with smaller strengths.
- The parameter *β_i_* in the outcome model (Equation 1) is more susceptible to the estimation bias of *α_i_* (24), the magnitude of tuning parameter *λ*_1_*_β_*, and the outcome noise (*ζ*), leading to higher MSE of the parameter ***β*** than ***α***. Shrinking the size of $\lambda_{1\beta }$ increases the likelihood of detecting nonzero *β_i_* values, thus improving the identification of mediators with nonzero effects.

### 3.4 Evaluation of models

Tuning Parameters: We selected seven values for the tuning parameters $\lambda_{1\alpha }$ and $\lambda_{1\beta }$: 0.001, 0.01, 0.1, 1, 2, 5, and 10. This resulted in 49 combinations of penalty parameters ( $\lambda_{1\alpha },\lambda_{1\beta }$) being examined. Additionally, we varied $\lambda_{\gamma }$ across a spectrum of 72 values, ranging from 0 to 100. Hence, we explored 3528 parameter combinations for ( $\lambda_{1\alpha },\lambda_{1\beta },\lambda_{\gamma }$), allowing for thoroughly exploring model behaviors under diverse settings.

Model Selection: To identify the optimal model, we utilized the Bayesian information criterion (BIC). BIC is calculated using the formula BIC=qln(n)−2ln(L), where *q* denotes the total number of nonzero estimated parameters and *L* is the estimated maximum likelihood. We opted for BIC instead of cross-validation (CV) for the following reasons. First, when a large penalty is applied, all parameter estimates may be reduced to zero. Consequently, the average predictive error would be solely determined by noise, potentially resulting in the lowest predictive error. This situation could lead to the incorrect identification of the optimal model. Additionally, CV is more computationally intensive. Therefore, choosing BIC is not only theoretically and statistically justified, but it is also practically meaningful.

Performance Metrics: We evaluated the performance of the estimated IE using TPR and True Negative Rate (TNR) to assess sensitivity and specificity, respectively. Mean Squared Error (MSE) and Relative Bias (RB) measured parameter estimation accuracy for $\hat{\alpha }$, $\hat{\beta }$, and $\hat{\alpha_{i}}\hat{\beta_{i}}$. Overall performance was assessed using Youden’s J statistic ( J=TPR+TNR−1), which combines sensitivity and specificity

### 3.5 Screening procedure for ultra-high dimensional mediators

To enhance the accuracy of parameter estimation and reduce computational load, we adopt SIS approach, which preserves essential information during the dimensional reduction process. In mediation analysis, SIS selects potential mediators based on their marginal correlations with the outcome. The reduced dimension is typically set at d=k[n/log(n)]. We hypothesize that the choice of *k* will be influenced by the ratio of the number of mediators to the sample size. We conduct a simulation study to identify the optimal value of *k*, particularly in cases where the number of mediators changes while the sample size remains fixed.

### 3.6 An algorithm for optimization and development of R packages

To estimate parameters, we employed the Alternating Direction Method of Multipliers (ADMM) (29) for the optimization problem. We have formulated an R package for the context of a high dimensional mediation model, designated as HDMAADMM (30), which implements ADMM with an SIS option for regularization. To expedite the large-scale computational tasks, we created two R packages, oneMKL and oneMKL.MatrixCal (31). These are designed to work seamlessly with the Intel oneAPI Math Kernel Library, offering the functions of matrix operations and parallel computing in R on both Linux and Windows platforms.

## 4 Simulation study

This section presents two simulation studies evaluating the effectiveness of our proposed tuning parameter strategies.

### 4.1 Designs for simulations

We simulated 200 independent and identically distributed samples. Each sample contained *n* = 50 subjects with a varying number of mediators *p* = 30, 50, 100, 150, and 200. The treatment exposure *X* was created using a Bernoulli distribution with a probability of 0.5. Given that our main purpose was to estimate IE, we set the true value for DE parameter *γ* to a relatively small value at 2. The parameters associated with $IE_{i}$ is *α_i_β_i_* organized as follows:

- The vector ***α*** = (*α*_1_*,α*_2_*,…,α_p_*), representing the effect of treatment on the mediator, had its first 20% of values as nonzeros, and the remaining 80% were set at zero.
- The vector ***β*** = (*β*_1_*,β*_2_*,…,β_p_*), indicating the effect of the mediator on the outcome, was arranged differently. The first 10% of parameters were given nonzero values, followed by 10%, which were set to zero. The next 10% were given nonzero values, and the remaining 70% were set at zero.
- For nonzero values of parameters *α_i_* and *β_i_*, we categorized them into large signals (constituting 5%, with a mean of 6 and a standard deviation of 0.1) and small-signals (constituting 5%, the mean being 4 and the standard deviation of 0.1). Both types of signals adhered to normal distributions.

Each mediator $M_{i}$ (for i=1,2,…,p) was derived using Equation 1, where the error distribution for the mediators adhered to a multivariate normal distribution with a mean vector of 0. For simplicity, the covariance matrix of mediators was defined as a compound symmetry matrix, with diagonal elements assigned to a value of 1 and off-diagonal elements assigned to another value of 0.1. This specification was informed by detecting very weak correlations among the FC mediators observed in the IP study. Given that IE for the ith mediator is defined as $IE_{i}=\alpha_{i}\beta_{i}$, we categorized mediators into four distinct types based on the nonzero status of their component parameters. Type 1 mediators (10%) exhibit nonzero IE ( $\alpha_{i}\neq 0$, $\beta_{i}\neq 0$), creating an X−M−Y pathway. The remaining 90% can be termed “zero-effect mediators”: Type 2 ( $\alpha_{i}=0$, $\beta_{i}\neq 0$) creates an M−Y pathway, Type 3 ( $\alpha_{i}\neq 0$, $\beta_{i}=0$) forms an X−M pathway, and Type 4 ( $\alpha_{i}=0$, $\beta_{i}=0$) shows no indirect effect.

The outcome variable *Y* is generated using Equation 1, where its error distribution follows a normal distribution with a mean of zero and a standard deviation of 0.1. To avoid estimation problems, particularly for mediators with small nonzero signals, we ensured that the standard deviation of the error for the outcome and the standard deviation of the nonzero parameter *α_i_* and *β_i_* were on the same scale. This approach helps get accurate estimates.

### 4.2 Simulation results

#### 4.2.1 Overestimation of DE

We initiate our analysis by examining the effectiveness of our first tuning parameter strategy in mitigating the overestimation of DE. In the simulation study, we compare LASSO that utilizes three distinct tuning parameter strategies, specifically, (1) $TR_{L}$: traditional LASSO method, without applying any penalty to DE (where the tuning parameter $\lambda_{\gamma }=0$), (2) $MD_{L}$: LASSO with a modified $L_{1}$-norm penalty $\lambda_{\gamma }\left|\gamma \right|$ (where $\lambda_{\gamma }>0$) for DE, and (3) $SMD_{L}$: LASSO with a strictly modified $L_{1}$-norm penalty $\lambda_{\gamma }\left|\gamma \right|$ (where $\;\lambda_{\gamma }\geq c$) for DE. This comparison examines how various tuning parameter strategies impact the accuracy of estimating parameters. Additionally, we report the findings by altering the number of mediators across various levels. The optimal model is determined by BIC.

An example illustrates the overestimation of DE when the number of mediators is 200. In this case, we assume the true value of TE to be 521.736, which consists of a DE of 2 and an IE of 519.736. Using the *TR_L_* strategy, the average estimation of DE tends to be greatly overestimated, reaching 505.382. IE is substantially underestimated at 12.998, leading to a poor TPR of IE (18.6%).

The *MD_L_* strategy significantly reduces the overestimation of DE to 312.117. Even though this estimate is substantially lower than the overestimation observed by the *TR_L_* strategy, it is still undesirable as the true value of DE is 2. The estimated IE increases from 12.998 to 194.406. The MSE of IE decreases from 82.094 to 68.408. The RB is also reduced, moving from -0.975 to -0.626, and the TPR of IE increases from 18.6% to 46.4%.

Among the three tuning parameter strategies evaluated, the overall performance of the *SMD_L_* strategy demonstrates superior overall performance compared to the alternative approaches. Using grid search, when threshold *c* is set to 0.3, the *SMD_L_* strategy reduces the estimated DE to 1.688, closely approximating the true DE value of 2. As a result, the *SMD_L_* strategy elevates the TPR of IE to 81.1%, reduces the MSE to 64.690, and lowers the RB to -0.059. Thus, adjusting the range of the tuning parameter *λ_γ_* beyond 1 is crucial for achieving the desired level of TPR of IE in high-dimensional mediation models.

**Figure 1:**
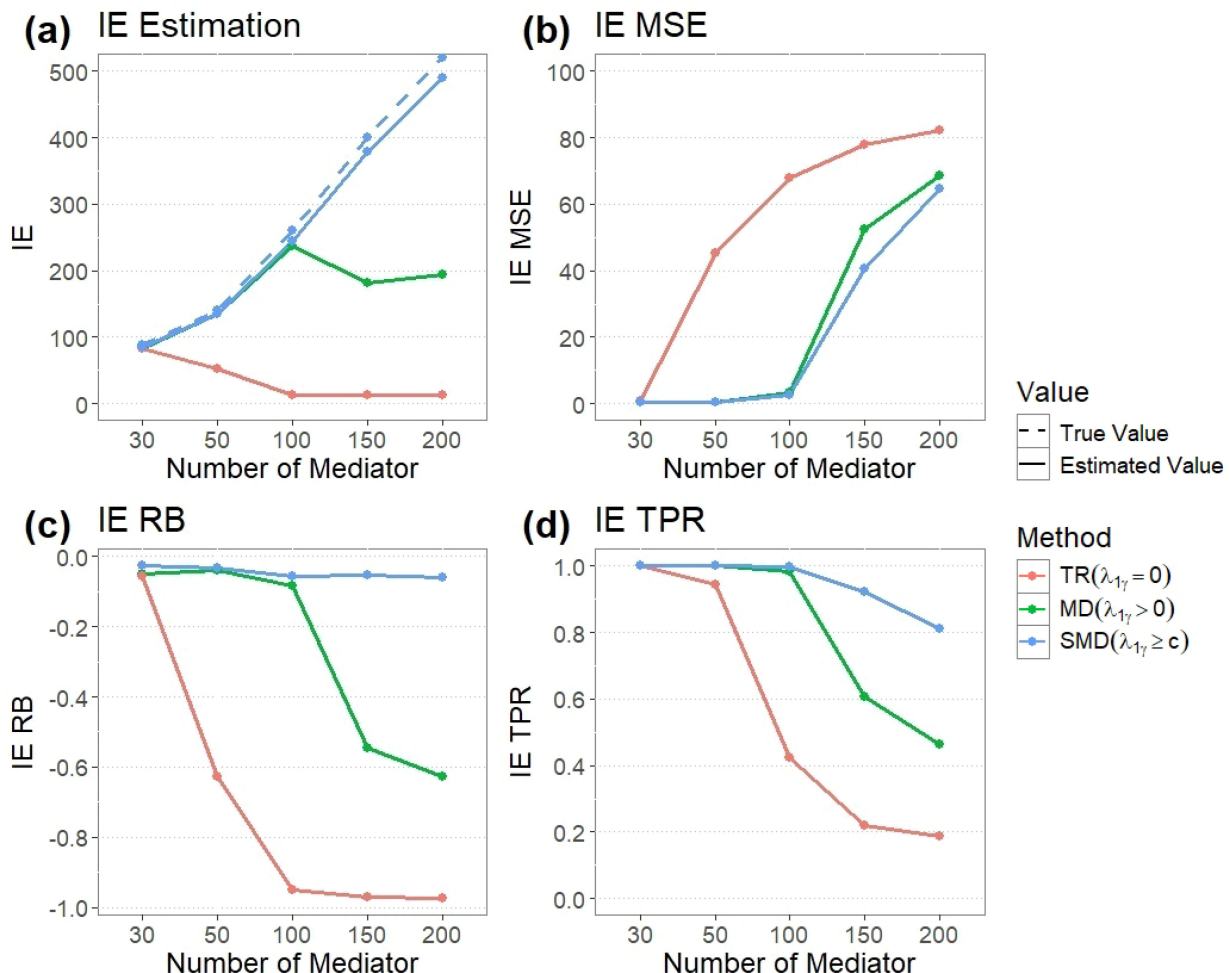
Simulated results by LASSO - overestimation of DE. **(a)** True and estimated IE values for methods TR, MD, and SMD for different number of mediators. **(b)** MSE of estimated IE for TR, MD, and SMD methods with different number of mediators. **(c)** RB of estimated IE for TR, MD, and SMD methods with different number of mediators. **(d)** TPR of estimated IE for TR, MD, and SMD methods with different number of mediators.

**Figure 1** presents simulation results with varying numbers of mediators. In a low-dimensional model setting (with *n* = 50 and *p* = 30), the *TR_L_* strategy accurately estimates IE. However, as the number of mediators increases, DE obtained by the *TR_L_* strategy is substantially overestimated. In high-dimensional settings (with *n* = 50 and *p* ≥ 50), the impact of the tuning parameter strategy on the estimation is consistent with the previous example (*p* = 200). The *TR_L_* strategy overestimates DE substantially, resulting in an estimation of IE close to zero, a larger estimation of MSE and RB, and a smaller estimation of TPR and TNR. Conversely, the *MD_L_* and *SMD_L_* strategies successfully mitigate this overestimation, enhancing the accuracy of estimation and biomarker detection. Notably, the *SMD_L_* strategy is particularly beneficial in estimating IE with desirable MSE, RB, TPR, and TNR.

The abovementioned findings assumed a small true value of 2 for DE. To further examine the *SMD_L_* strategy, we conducted another simulation study with a sample size of 50 and 200 mediators, mainly focusing on situations with larger DE values. In this simulation, the true value of IE was fixed at 320, while the true values of DE were varied across a spectrum of 36, 80, 137, 213, 320, 480, 747, 1280, 2880, and 6080. This spectrum represents a transition in the mediation proportion (i.e., the ratio of IE to TE) from 90% to as low as 5%.

Simulation results for the *MD_L_* and *SMD_L_* strategies showed an overestimation bias in IE, intensifying as the true value of DE increased. The underlying reason for this phenomenon is that the TE is a sum of DE and TIE; when the estimation of DE is constrained, the estimation of TIE correspondingly increases, leading to a rise in both the MSE and RB of IE. Moreover, a threshold *c* in the *SMD_L_* strategy was ascertained by identifying the minimal MSE of DE derived from the grid research outcomes. This strategy facilitates an accurate estimation of DE and enhances the TPR of IE.

It is noteworthy that the TPR of IE under the *SMD_L_* strategy ranges between 69% and 81%, which is significantly larger compared to the *MD_L_* strategy (ranging from 46% to 60%) and the *TR_L_* strategy (ranging from 16% to 27%). The TNR of IE for the three strategies exhibits similar values. To conclude, when the research objective is to enhance biomarker detection rather than providing a precise estimate of IE, the *SMD_L_* strategy remains an effective strategy for addressing the problem of overestimation of DE.

#### 4.2.2 Improving the detection rate of nonzero-effects of mediators

Another simulation study examined whether the second tuning parameter strategy further improves model performance. We relaxed the assumption of the *L*_1_-norm equal-sized tuning parameter constraint (where *λ*_1_*_α_*= *λ*_1_*_β_*) for parameters regarding IE (i.e., ***α*** and ***β***) and observed whether this relaxation contributes to the improvement of model performance.

**Table 1** illustrates the impact of this relaxation of the constraint using an example with 20 nonzero mediators, with the estimation of parameters obtained via the *SMD_L_* strategy. When penalty sizes are equal, such as *λ*_1_*_α_*= 1 and *λ*_1_*_β_*= 1, we observe that only 12 out of 20 nonzero mediators are detected, yielding a TPR of IE of 60%. Reducing *λ*_1_*_α_* to 0.1 while keeping *λ*_1_*_β_* at 1 (where *λ*_1_*_α_< λ*_1_*_β_*) does not improve the TPR of IE, as it does not enhance the TPR of ***β***. However, lowering *λ*_1_*_β_* to 0.01 while keeping *λ*_1_*_α_* at 1 leads to the detection of 10 additional nonzero *β_i_*, raising the TPR of IE to 95%.

**Table 1:** Performance of *SMD_L_*under various combinations of *λ*_1_*_α_*and *λ*_1_*_β_*.

Tuning parameter strategy					$\lambda_{1\alpha \;}=\;\lambda_{1\beta }$			$\lambda_{1\alpha \;}<\;\lambda_{1\beta }$			$\lambda_{1\alpha \;}>\;\lambda_{1\beta }$		
					$\left(\lambda_{1\alpha }=1,\;\lambda_{1\beta }=1\right)$			$\left(\lambda_{1\alpha }=0.1,\;\lambda_{1\beta }=1\right)$			$\left(\lambda_{1\alpha }=1,\;\lambda_{1\beta }=0.01\right)$		
Signal	Mediator	True value			Estimation								
		*α*	*β*	*IE*	*α*	*β*	*IE*	*α*	*β*	*IE*	*α*	*β*	*IE*
Large	1	6.073	5.936	36.049	6.115	4.901	29.971	6.236	4.901	30.562	6.115	**4.029**	24.634
	2	6.141	6.016	36.944	5.780	1.696	9.804	5.894	1.696	9.997	5.780	**3.583**	20.711
	3	5.912	5.823	34.426	5.515	0.000	0.000	5.626	0.000	0.000	5.515	**0.632**	3.486
	4	5.899	5.758	33.968	5.869	0.000	0.000	5.987	0.000	0.000	5.869	**1.099**	6.452
	5	6.160	5.827	35.898	5.606	0.000	0.000	5.718	0.000	0.000	5.606	**3.539**	19.841
	6	6.168	5.853	36.101	6.065	9.064	54.975	6.185	9.064	56.057	6.065	**5.618**	34.074
	7	5.929	5.913	35.054	5.992	10.494	62.881	6.111	10.494	64.131	5.992	**4.430**	26.547
	8	6.091	5.989	36.482	5.667	12.946	73.361	5.779	12.946	74.811	5.667	**13.882**	78.666
	9	6.084	5.977	36.364	6.206	9.021	55.989	6.329	9.021	57.092	6.206	**7.999**	49.644
	10	5.850	5.922	34.644	6.024	0.000	0.000	6.142	0.000	0.000	6.024	**0.456**	2.746
Small	11	4.009	3.968	15.907	4.026	0.144	0.580	4.111	0.144	0.593	4.026	**0.753**	3.032
	12	4.075	4.180	17.034	4.216	0.000	0.000	4.301	0.000	0.000	4.216	**2.936**	12.379
	13	4.188	4.061	17.007	4.043	2.021	8.171	4.125	2.021	8.336	4.043	**3.576**	14.457
	14	4.101	3.966	16.267	3.883	3.989	15.488	3.966	3.989	15.818	3.883	**2.553**	9.914
	15	3.934	3.892	15.315	3.894	5.549	21.607	3.975	5.549	22.060	3.894	**2.602**	10.131
	16	3.813	4.146	15.807	4.012	6.667	26.745	4.095	6.667	27.305	4.012	**5.908**	23.701
	17	4.123	3.949	16.280	3.678	0.000	0.000	3.756	0.000	0.000	3.678	**0.000**	0.000
	18	4.023	3.935	15.832	4.313	0.000	0.000	4.403	0.000	0.000	4.313	**1.898**	8.184
	19	4.130	4.171	17.224	4.071	4.596	18.709	4.159	4.596	19.113	4.071	**2.473**	10.067
	20	3.740	3.953	14.783	4.022	0.000	0.000	4.107	0.000	0.000	4.022	**1.003**	4.035
IE Performance		MSE			0.060	5.280	71.666	0.070	5.280	73.887	0.060	3.470	**47.907**
		TNR			0.138	0.969	0.972	0.013	0.969	0.972	0.175	0.819	**0.806**
		TPR			1.000	0.300	0.600	1.000	0.300	0.600	1.000	0.800	**0.950**

Bold values indicate results when *λ*_1*α*_=1 and *λ*_1*β*_ = 0.01 (i.e., *λ*_1*α*_ > *λ*_1*β*_), showing more nonzero *β* estimates and improved performance (lower MSE and higher TPR) compared with the other two scenarios.

Given that the sum of DE and TIE is a fixed number (TE), the accuracy of estimating DE will inherently influence the estimation outcomes of IE. Consequently, the first tuning parameter strategy (*λ_γ_*|*γ*| where *λ_γ_*≥ *c*), designed to determine the estimation of DE, needs to be taken into account when evaluating the second strategy. Thus, a total of 8 methods were compared in this simulation study based on the variants of Pathway LASSO LASSO.

Four variants of Pathway LASSO method were implemented: *SMD.S_p_* (where $\lambda_{\gamma }\geq c$, $\lambda_{1\alpha }>\lambda_{1\beta }$), *SMD.E_p_* ( $\lambda_{\gamma }\geq c$, $\lambda_{1\alpha }=\lambda_{1\beta }$), *MD.S_p_* ( $\lambda_{\gamma }>0$, $\lambda_{1\alpha }>\lambda_{1\beta }$), and *MD.E_p_* ( $\lambda_{\gamma }>0$, $\lambda_{1\alpha }=\lambda_{1\beta }$), with traditional Pathway LASSO equivalent to *MD.E_p_*. For a consistent comparison, the tuning parameters κ and ν in the path-specific penalty $P_{1}\left(\alpha ,\beta \right)=\kappa \sum_{i=1}^{p}\left[\left|\alpha_{i}\beta_{i}\right|+\nu \left(\alpha_{i}^{2}+\beta_{i}^{2}\right)\right]$ were fixed across all methods. The parameter κ, crucial in determining the magnitude of estimated IE, was set to 0.01 to optimize results, leading to higher TPR and lower MSE of IE, as well as reduced BIC of the model. The parameter ν was fixed at 2, adhering to recommendations from traditional Pathway LASSO. Similarly, another four methods are implemented on LASSO, henceforth referred to as *SMD.S_L_, SMD.E_L_, MD.S_L_*, and *MD.E_L_*, respectively.

To evaluate the performance of the proposed model for the aforementioned methods, we refrained from identifying an optimal model among the 3,528 tuning parameter combinations ( $\lambda_{\gamma },\lambda_{1\alpha },\lambda_{1\beta }$). We noted the potential bias induced by the substantial variance in BIC across various penalty configurations ( $\lambda_{1\alpha },\lambda_{1\beta }$). Therefore, we employed a two-stage model selection process. The first stage involves selecting the optimal model based on BIC for each sample, considering different penalty configurations ( $\lambda_{1\alpha },\lambda_{1\beta }$). The second stage aggregated the results by computing the average performance across the collection of the optimal model and penalty configurations within each method. This selection method aligned with the Minimax property principle, which ensured a more robust and reliable assessment of model performance.

**Figure 2:**
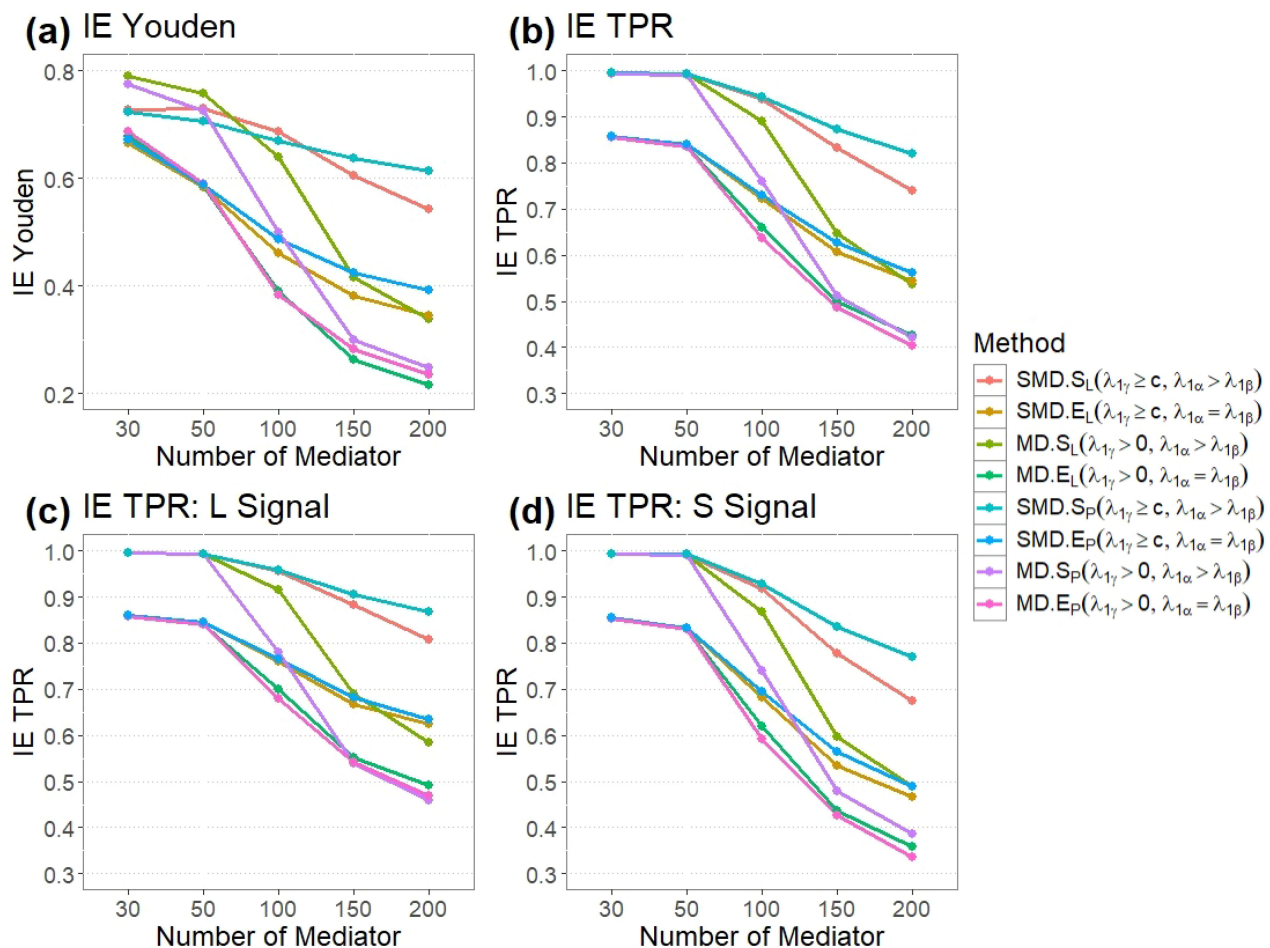
A Comparison between Pathway LASSO and LASSO for various combinations of *λ*_1_*_α_*and *λ*_1_*_β_*. **(a)** Youden index for IE estimation for 8 methods with different numbers of mediators. **(b)** TPR for IE estimation for 8 methods with different numbers of mediators. **(c)** TPR for IE estimation, when signal is large, for 8 methods with different numbers of mediators. **(d)** TPR for IE estimation, when signal is small, for 8 methods with different numbers of mediators.

**Figure 2** displays the simulation results with varying numbers of mediators. These results indicate that implementing our proposed tuning parameter strategies, *SMD.S_P_* and *SMD.S_L_*, exhibits superior performance. Specifically, as the number of mediators increases, *SMD.S_P_*, *SMD.E_P_*, *SMD.S_L_*, and *SMD.E_L_*, employing our first tuning parameter strategy for DE (i.e., setting $\lambda_{\gamma }\geq c$) show better performance compared to those that do not apply this strategy, which involves setting a constraint on the tuning parameter for IE (i.e., setting $\lambda_{1\alpha }>\lambda_{1\beta }$).

We further evaluated the effectiveness of the second tuning parameter strategy in enhancing model performance. Based on the simulation results for a scenario involving 200 mediators, we can infer that the methods implemented with a smaller size of the tuning parameter $\lambda_{1\beta }$, (specifically, *MD.S_p_, SMD.S_p_, MD.S_L_* and *SMD.S_L_*), demonstrate a better model performance. This improvement is evident showing smaller MSE compared to cases where the penalty size for $\lambda_{1\alpha }$ and $\lambda_{1\beta }$ is equal (namely, methods *MD.E_p_, SMD.E_p_, MD.E_L_* and *SMD.E_L_*). This finding suggests that our second tuning parameter strategy, which includes reduced $\lambda_{1\beta }$, tends to yield estimates (particularly for parameters β) that are closer to the corresponding actual model parameters. It accurately identifies the mediators with nonzero-effects. Our proposed method, *SMD.S_p_*, outperforms others across IE metrics, with the highest TPR (81.9%), F1 score (0.504), Youden Index (0.614), and lowest MSE (45.240). For signal detection, *SMD.S_p_* excels with TPRs of 86.7% for large signals and 77.1% for small signals, surpassing other methods in identifying both magnitudes.

#### 4.2.3 Sure independence screening for finding optimal scaling factor

In this section, we conduct a simulation study to test our hypothesis that the choice of the scaling factor *k* in SIS procedure is influenced by the ratio of the number of mediators (*p*) to the sample size (*n*), referred to as the *p/n* ratio. For this simulation, we keep the sample size constant at 50 while changing the number of mediators to 200, 500, and 1000. This gives us *p/n* = 4, 10, 20. We calculated the reduced dimension as *d* = *k*[*n/log*(*n*)], and adjusted the scaling factor *k* across a range of values: 0, 1, 2, 3, 4, 5, 6, 7, 8, 9, 10, 15, 20, 30, and 40.

**Figure 3:**
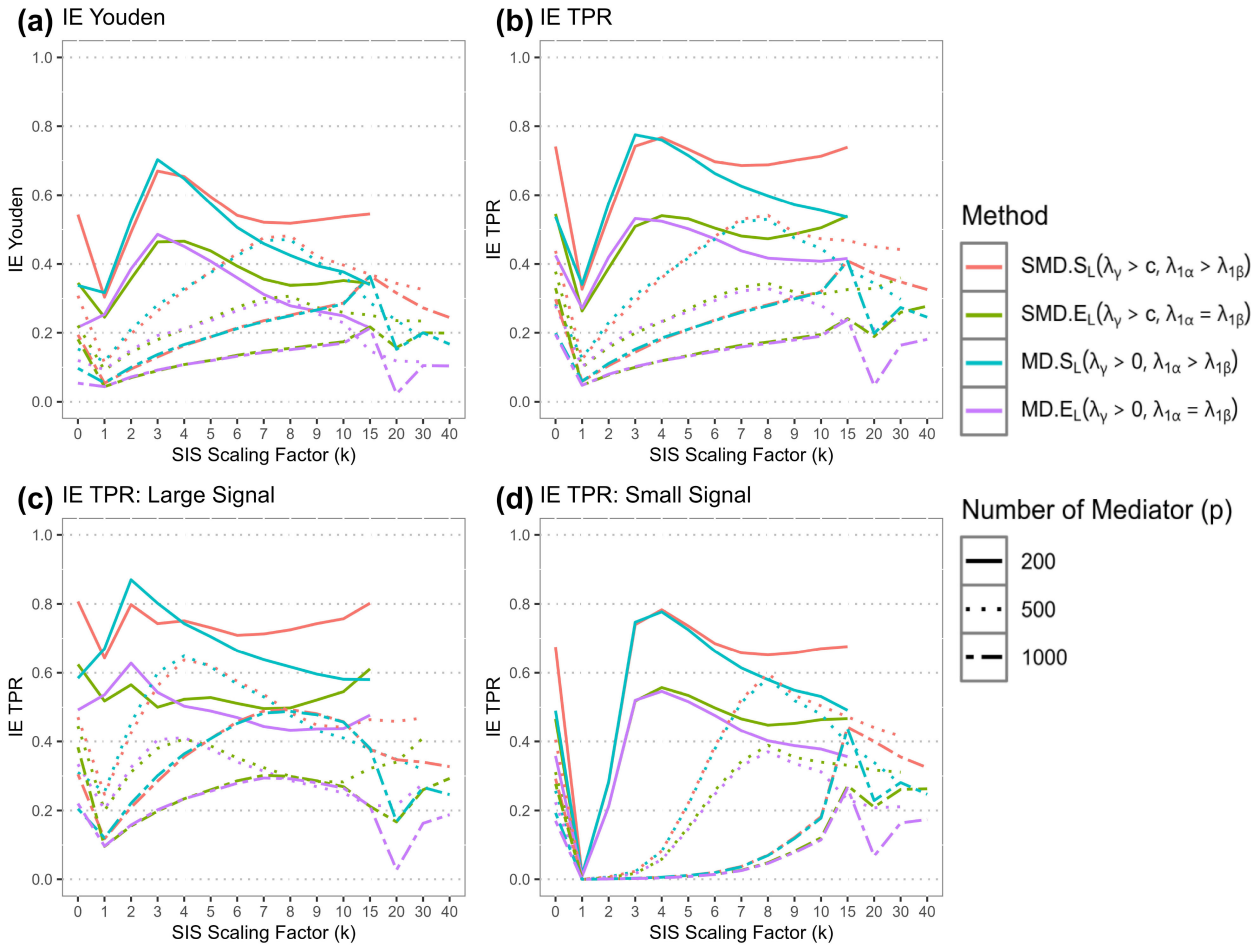
Simulated results for SIS. **(a)** IE Youden index for 4 methods under different SIS scaling factors; line types denote numbers of mediators. **(b)** IE TPR for 4 methods under different SIS scaling factors. **(c)** IE TPR for 4 methods, when signal is large, under different SIS scaling factors. **(d)** IE TPR for 4 methods, when signal is small, under different SIS scaling factors.

**Figure 3** presents simulated results for the varying scaling factor *k*. When the number of mediators was set at 200, yielding a *p/n* ratio to 4, the largest TPR for identifying mediators with nonzero effects is attained at *k* = 3 and 4. This corresponds to reduced dimensions of 38 and 51, respectively. When the number of mediators is increased to 500, leading to a *p/n* ratio of 10, the TPR peaks at *k* = 8, giving a reduced dimension of mediators at 102. In the case of 1000 mediators, where the *p/n* ratio stands at 20, the best TPR occurs at *k* = 15 or *k* = 20, resulting in reduced dimensions of 192 or 256, respectively. These findings support our proposition that the *p/n* ratio influences the optimal choice of *k*. The understanding of the motivational forces behind this is that when the reduced dimension *d* is increased, the sure screening property of SIS method is more likely to capture a greater number of true signals. Based on the findings from our simulations, with a fixed sample size of *n* = 50, it is advisable for researchers to select the *k* value in accordance with the *p/n* ratio.

#### 4.2.4 Model comparison

**Figure 4:**
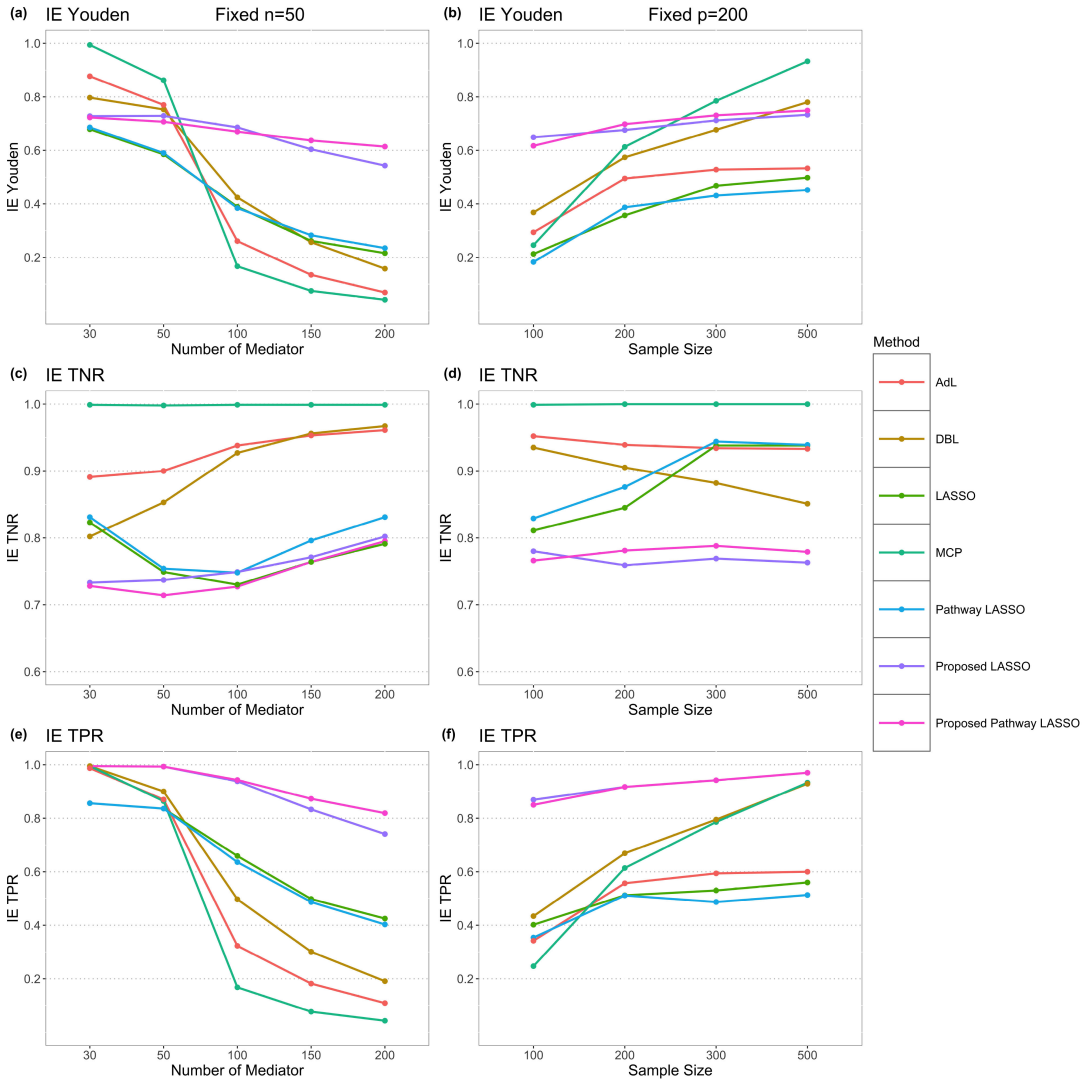
Model comparison. **(a, c, e)** Youden index, TNR, and TPR for IE estimation using 7 methods under different numbers of mediators, when sample size n fixed at 50. **(b, d, f)** Youden index, TNR, and TPR for IE estimation using 7 methods under different sample sizes, when the number of mediators p fixed at 200.

We evaluated seven regularization approaches: the proposed LASSO (*SMD.S_L_*), the proposed Pathway LASSO (*SMD.S_P_*), LASSO, Pathway LASSO, MCP, DBL, and AdL. Simulations were conducted under both small and large sample scenarios. In the small sample setting, the sample size was fixed at *n* = 50 and the number of mediators (*p*) varied from 30, 50, 100, 150, to 200. In the large sample setting, *p* was fixed at 200 and *n* varied from 50, 100, 200, 300, to 500. **Figure 4** presents the model performance in term of Youden Index, TNR, and TPR.

In terms of TPR, the proposed LASSO and proposed Pathway LASSO substantially outperformed the other five approaches for both small sample and large sample scenarios. In a small sample case with *n* = 50 and *p* = 200, their TPRs were 0.741 and 0.819, compared with 0.425 for LASSO and 0.403 for Pathway LASSO. The TPR of IE for MCP, DBL, and AdL were only 0.043, 0.191, and 0.109, respectively. For a large sample case with *n* = 500 and *p* = 200, the TPRs of both proposed LASSO and proposed Pathway LASSO reached 0.970, slightly larger than MCP (0.933) and DBL (0.929). In contrast, LASSO, Pathway LASSO, and AdL identified only half of the true signals, with TPRs ranging from 0.513 to 0.600.

In evaluating Youden Index, our proposed LASSO and Pathway LASSO consistently ranked among the top four methods, effectively balancing TPR and TNR across both small and large sample scenarios. This reflects their superior ability to detect true signals while correctly excluding non-signals. Although Pathway LASSO achieved slightly better TNR than our approaches, this improvement came at the expense of markedly lower TPR, resulting in Youden indices that did not exceed 0.452, even under large-sample conditions.

MCP and DBL performed best in large sample, low dimensional settings (e.g., *p* = 200 with *n* = 300 or 500), where their Youden indices surpassed those of our proposed methods. However, in small sample scenarios, both methods produced the poorest outcomes in terms of both Youden index and TPR. In contrast, our proposed LASSO and Pathway LASSO consistently delivered the strongest performance in high-dimensional settings (i.e., *n < p*), across both small and large sample cases, underscoring their robustness and applicability to high-dimensional models

## 5 A real world application

We applied our proposed approach, denoted as *SMD.S_p_*(symbolizing the application of Pathway LASSO integrated with the two proposed tuning parameter strategies), to identify FC mediators with nonzero IE in the IP and LLD study, respectively.

### 5.1 The IP study

The primary objective of the IP study is to elucidate the mediating role of FC between anti-depressant treatment and the alterations in neurobehavioral outcomes. To improve the TPR of IE and ensure computational feasibility, the SIS framework was implemented, utilizing a scaling factor of *k* = 15. This adjustment significantly decreased the number of FC mediators we examined from 5460 to 206, aligning with the common practice of assuming signal sparsity at 95-98%. Through the screening procedure, the remaining FC revealed a much stronger association between the improvement in DASS scores and changes in FC within the IP group, taking into account the baseline FC levels of the HC group.

**Figure 5:**
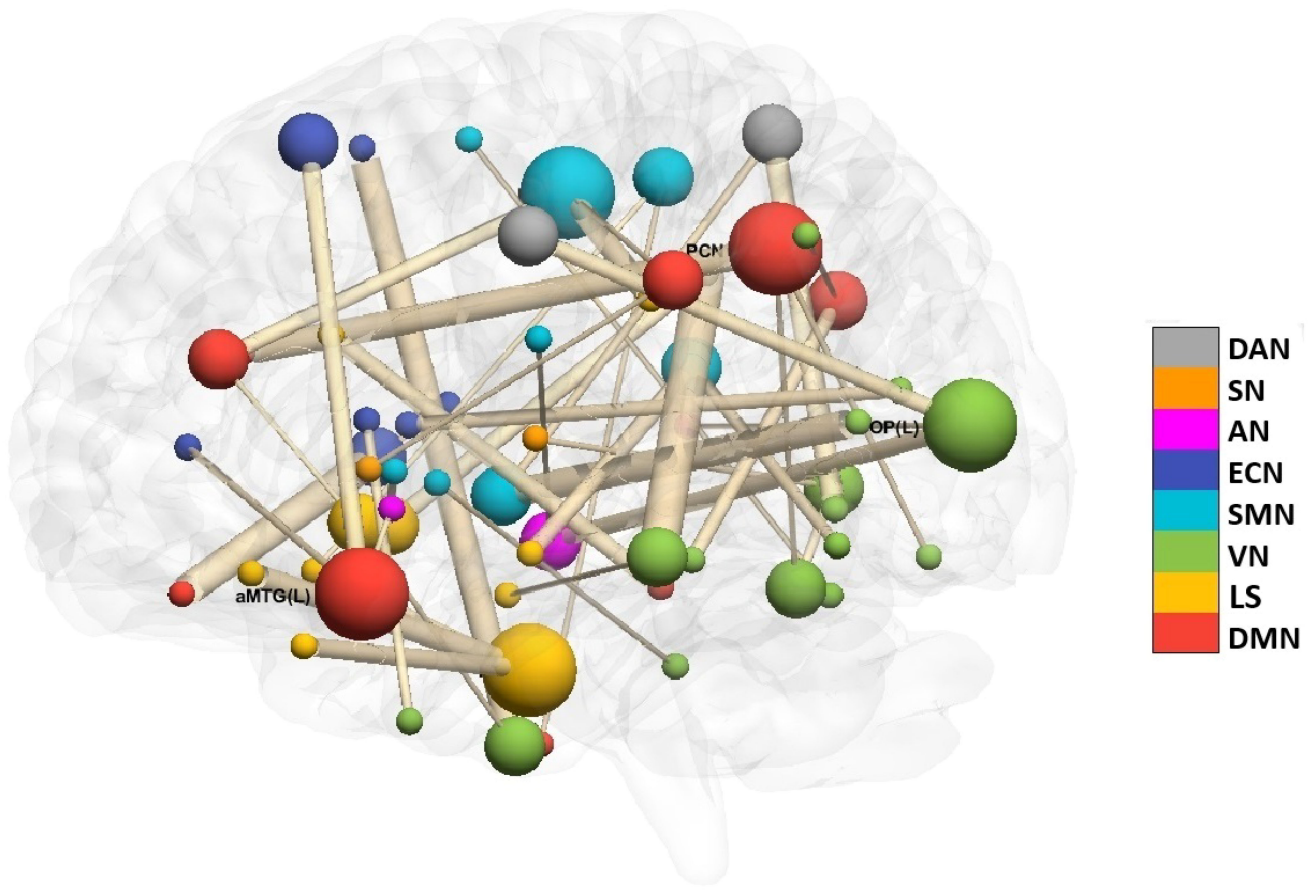
All 43 identified links - IP study.

Of the 206 FC links, only 43 FC mediators exhibited a nonzero IE (see **Figure 5**). In this figure, nodes represent ROIs, with larger nodes indicating hubs (i.e., ROIs with more connected FC links). The lines between nodes represent FC links, with line thickness reflecting the strength of IE. Based on the biomarker detection results, the therapeutic application of SSRIs may alleviate the aberrations observed in these identified FC mediators among individuals with IP which exhibit cognitive deficits, potentially reducing their symptoms. The top 10 FC mediators with nonzero IE are presented in **Table 2**. The ROIs associated with the top identified FC mediators align with previous research findings by demonstrating a pronounced association with the symptoms of IP, notably depression and anxiety.

**Table 2:** TOP 10 identified links - IP study.

Rank	ROI 1		ROI 2		IE	$\hat{a}$	$\hat{\beta }$	IP-SSRI			HC			SSRI vs. HC
	Name	Network	Name	Network				Pre	Post	Change	Pre	Post	Change	Change
1	aSMG(L)	DAN	toITG(L)	VN	-1.321	-0.132	10.025	0.467	0.312	-0.154	0.281	0.307	0.026	-0.181
2	FMC	DMN	IFGtr(R)	ECN	-1.074	0.155	-6.934	-0.075	0.020	0.095	-0.008	-0.113	-0.105	0.200
3	TFa(R)	VN	SFG(R)	ECN	-0.978	0.139	-7.059	-0.125	0.052	0.177	0.036	0.036	0.000	0.177
4	PAL(R)	SMN	CALC(L)	VN	-0.977	0.097	-10.123	0.063	0.066	0.002	0.073	-0.056	-0.129	0.131
5	PCN	DMN	PAC(R)	DMN	-0.918	0.104	-8.821	0.119	0.180	0.061	0.212	0.132	-0.080	0.141
6	PHa(R)	LS	FOC(L)	LS	-0.788	-0.155	5.072	0.165	0.033	-0.132	0.001	0.067	0.066	-0.198
7	LING(R)	VN	SPL(R)	DAN	-0.763	0.068	-11.262	-0.058	-0.009	0.049	0.045	-0.014	-0.059	0.108
8	PCC	DMN	PRG(R)	SMN	-0.751	-0.110	6.835	-0.137	-0.206	-0.070	-0.244	-0.161	0.083	-0.152
9	PHa(R)	LS	TP(L)	LS	-0.647	-0.145	4.456	0.466	0.328	-0.138	0.269	0.320	0.051	-0.189
10	aMTG(L)	DMN	SFG(L)	ECN	-0.632	-0.083	7.578	0.101	0.067	-0.034	0.112	0.202	0.090	-0.124

For instance, the FC mediator with the most substantial IE effect (-1.321) is situated between two specific ROIs: the left anterior division of the supramarginal gyrus (SMG, BA 40) and the left temporooccipital part of the inferior temporal gyrus (toITG, BA 20). SMG, an integral component of the DAN, is implicated in top-down control of attention. Abnormality in SMG has been observed in individuals diagnosed with LLD or social anxiety disorder (SAD) (32, 33). In addition, the inferior temporal gyrus, a constituent of the visual network, plays a vital role in cognitive processes such as visual processing, object recognition, and semantic processing. Dysfunction in ITG has been identified and demonstrated to be associated with SAD, MDD, and LLD (34, 35).

The FC mediator with the second most substantial IE (-1.074) is identified between the frontal medial cortex (FMC) and the right pars triangularis of the inferior frontal gyrus (IFGtr, BA 45). The FMC, part of the DMN, includes the medial prefrontal cortex (mPFC) and ventral medial prefrontal cortex (vmPFC). It plays a crucial role in various cognitive functions, mood regulation, and self-referential tasks. The IFGtr, part of the ECN, is involved in higher-order cognitive processes. Both ROIs are recognized in the literature as critical areas associated with IP symptoms (36–40).

### 5.2 LLD study

The primary objective of LLD study is to distinguish brain patterns between LLD and HC groups based on whole-brain FC alterations. The SIS framework was implemented with a scaling factor of *k* = 29, which reduced the number of examined FC mediators from 3741 to 190, consistent with the common assumption of signal sparsity at 95%.

Of the 190 FC links, only 18 FC mediators exhibited nonzero IEs. The top 10 FC mediators are listed in **Table 3**. These ROIs exhibit FC patterns consistent with prior studies implicating them in LLD. For example, the FC mediator with the most substantial IE effect (0.476) is situated between two specific ROIs: the left hippocampus (HP) and the left fusiform gyrus (FG, BA 37, 20). HP is a core limbic system structure essential for memory formation and emotional regulation. FG is implicated in high-level visual processing, including face and object recognition. Abnormality in HP or FG has been observed in individuals diagnosed with LLD (33, 39, 41–44).

**Table 3:** TOP 10 identified links - LLD study.

Rank	ROI 1		ROI 2		IE	$\hat{a}$	$\hat{\beta }$	LLD	HC	LLD vs. HC
	Name	Network	Name	Network						Change
1	HP(L)	LS	FG(L)	VN	0.476	0.098	4.841	0.397	0.284	0.113
2	MTG(L)	DMN	STG(L)	AN	0.343	-0.229	-1.496	0.413	0.673	-0.260
3	ENT(L)	DMN	ITG(R)	VN	0.342	-0.237	-1.444	-0.079	0.181	-0.260
4	rACC(L)	DMN	TP(R)	LS	0.275	-0.115	-2.391	0.009	0.144	-0.135
5	PO(L)	CEN	RMF(R)	CEN	0.248	-0.174	-1.424	0.008	0.202	-0.194
6	VDC(L)	LS	rACC(L)	DMN	0.240	-0.145	-1.653	-0.048	0.116	-0.164
7	cACC(R)	SN	PRG(R)	SMN	0.218	0.102	2.146	0.184	0.064	0.120
8	PCC(L)	DMN	FP(L)	ECN	0.179	-0.132	-1.350	-0.019	-0.131	-0.150
9	ENT(R)	DMN	SMG(R)	DAN	0.125	-0.115	-1.081	-0.069	0.064	-0.133
10	CAU(R)	ECN	IC(L)	SN	0.121	-0.118	-1.022	0.032	0.169	-0.137

## 6 Conclusion and discussion

This work enhances biomarker detection in high-dimensional mediation models by introducing novel tuning parameter strategies for *L*_1_-norm penalties on DE and IE. The first strategy mitigates the overestimation of DE observed in conventional regularization methods such as Pathway LASSO, thereby enhancing the TPR of IE. The second strategy improves the ability to identify mediators with nonzero ***β***, leading to a better detection of biomarkers. Collectively, these strategies contribute to a robust and accurate analytical framework for navigating the complexities inherent in mediation analysis. Furthermore, we propose an optimal method for selecting the scaling factor within SIS procedure that significantly boosts TPR for mediators, especially in scenarios characterized by ultra high-dimensional mediators. Our proposed model is versatile and can be applied to various fields that deal with high-dimensional data, such as genomics, epidemiology, finance, and social sciences.

The current work applies the tuning parameter strategies to the single-modality mediation model involving FC. Investigating a multimodal approach integrating FC and SC may augment biomarker identification. Furthermore, this work primarily focuses on penalized point estimation, which yields a biased estimate of correlations among mediators. Alternative methodologies, such as debiased LASSO, permutation test, and bootstrapping technique, should be considered to assess the significance of nonzero-effect mediators. In addition, existing penalties including Pathway LASSO do not fully utilize brain network information. To better detect biomarker hubs for neuromodulation and faster recovery, future research could benefit from exploring penalty strategies such as network-constrained penalties (45).

## Data Availability

The original contributions presented in the study are included in the article/**Supplementary Material**. Further inquiries can be directed to the corresponding author.
