# Supplementary material for: A novel strategy for detecting multiple mediators in high-dimensional mediation models
Source: Front Psychiatry. 2025 Dec 15;16:1611761. doi: 10.3389/fpsyt.2025.1611761 (PMC12745155; doi:10.3389/fpsyt.2025.1611761)
Supplement: Supplementary file 1 [file Table1.pdf]

Supporting Information for "A Novel Strategy for Detecting Multiple Mediators in High-Dimensional Mediation Models"

Pei-Shan Yen[1], Zhaoliang Zhou[1], Soumya Sahu[1], Debarghya Nandi[1], Olusola Ajilore[2], and Dulal Bhaumik[1,2*]
[1]Division of Epidemiology and Biostatistics, University of Illinois at Chicago, Chicago, US
[2]Department of Psychiatry, University of Illinois at Chicago, Chicago, US
*email: dbhaumik@uic.edu

September 19, 2025

A The 105 brain regions of interest

The brain network was segmented into 105 ROIs based on the CONN atlas (Whitfield-Gabrieli and Nieto-Castanon, 2012). These ROIs include 50 pairs of bilateral regions and 5 central regions, as detailed in Web Table 1. In total, these ROIs account for 5460 unique FC (or links), calculated using the formula for combinations: $\binom{105}{2} = 105 \times 104/2$.

Web Table 1 also presents the functional networks as defined by Tessitore et al. (2019): auditory network (AN), central executive network (CEN), dorsal attention network (DAN), default mode network (DMN), salience network (SN), sensorimotor network (SMN), and visual network (VN). To comprehensively study IP patients, we have included the limbic system (LS) (Rajmohan and Mohandas, 2007).

References

Rajmohan, V. and Mohandas, E. (2007). The limbic system. *Indian Journal of Psychiatry* **49,** 132–139.

Tessitore, A., Cirillo, M., and De Micco, R. (2019). Functional connectivity signatures of parkinson's disease. *Journal of Parkinson's Disease* **9,** 637–652.

Whitfield-Gabrieli, S. and Nieto-Castanon, A. (2012). Conn: a functional connectivity toolbox for correlated and anticorrelated brain networks. *Brain Connectivity* **2,** 125–141.

Web Table 1: The 105 brain regions of interest in CONN atlas

Network[a]	ROI[b]	Region Name	Brodmann Area	Coordinates x	y	z
AN	H	Heschl's Gyrus	41,43	-45.2	-20.3	7.2
	PP	Planum Polare	38	-46.6	-6.0	-7.3
	PT	Planum Temporale	22	-52.7	-29.7	10.8
	aSTG	Superior Temporal Gyrus anterior division	22,41-42	-56.2	-3.9	-8.0
	pSTG	Superior Temporal Gyrus posterior division	22,41-42	-62.3	-29.2	3.8
DAN	SPL	Superior Parietal Lobule	5,7	-29.3	-49.5	57.5
	aSMG	Supramarginal Gyrus anterior division	40	-56.8	-32.8	37.2
DMN	AG	Angular Gyrus	39	-50.4	-55.7	29.7
	FMC	Frontal Medial Cortex	10-12, 24-25, 32-33	-4.9	43.4	-18.2
	aITG	Inferior Temporal Gyrus anterior division	20	-48.1	-5.0	-39.2
	aMTG	Middle Temporal Gyrus anterior division	21	-57.5	-4.2	-22.1
	PAC	Paracingulate Gyrus	32	-6.2	36.7	20.8
	PHp	Parahippocampal Gyrus posterior division	28, 34-36	-21.9	-32.4	-16.9
	PCN	Precuneous Cortex	7, 31	-7.7	-60.0	37.4
	pSMG	Supramarginal Gyrus posterior division	40	-54.9	-46.0	33.2
ECN	CAU	Caudate	-	-12.8	9.0	9.7
	FP	Frontal Pole	10	-24.7	53.0	7.5
	IFGop	Inferior Frontal Gyrus pars opercularis	44	-50.6	14.5	15.4
	IFGtr	Inferior Frontal Gyrus pars triangularis	45	-49.7	28.5	8.7
	MFG	Middle Frontal Gyrus	46	-38.1	18.4	42.1
	SFG	Superior Frontal Gyrus	8	-14.1	18.7	56.2
SMN	CO	Central Opercular Cortex	44,47	-48.0	-8.6	11.8
	SMA	Juxtapositional Lobule Cortex	6	-5.4	-2.8	56.1
	PAL	Pallidum	10	-19.0	-5.1	-1.3
	PO	Parietal Operculum Cortex	40,43	-48.4	-31.9	20.5
	poCG	Postcentral Gyrus	1,2,3	-38.4	-27.9	51.7
	PRG	Precentral Gyrus	4	-33.7	-11.8	49.4
	PUT	Putamen	-	-24.9	0.5	0.3
SN	FO	Frontal Operculum Cortex	6	-39.7	18.3	4.5
	IC	Insular Cortex	13-16	-36.4	1.2	0.1
	THL	Thalamus	-	-10.2	-19.3	6.3
VN	CN	Cuneal Cortex	17	-8.2	-80.3	27.1
	pITG	Inferior Temporal Gyrus posterior division	20	-53.4	-28.5	-26.0
	toITG	Inferior Temporal Gyrus temporooccipital part	20	-51.8	-53.4	-16.5
	CALC	Intracalcarine Cortex	17	-10.2	-75.0	8.0
	iLOC	Lateral Occipital Cortex inferior division	19	-45.1	-75.5	-1.9
	sLOC	Lateral Occipital Cortex superior division	19	-32.0	-72.9	38.0
	LING	Lingual Gyrus	17	-12.3	-65.7	-5.4
	pMTG	Middle Temporal Gyrus posterior division	21	-60.9	-27.4	-11.0
	toMTG	Middle Temporal Gyrus temporooccipital part	21	-57.6	-53.0	0.8
	OF	Occipital Fusiform Gyrus	37	-26.6	-76.6	-13.6
	OP	Occipital Pole	17-19	-16.9	-96.5	6.7
	SCLC	Supracalcarine Cortex	17,18	-2.1	-79.7	13.7
	TFa	Temporal Fusiform Cortex anterior division	37	-31.9	-4.4	-41.9
	TFp	Temporal Fusiform Cortex posterior division	37	-36.0	-29.5	-25.1
	TOF	Temporal Occipital Fusiform Cortex	37	-33.5	-53.7	-16.0
LS	Acb	Accumbens	-	-9.5	11.5	-7.2
	AMYG	Amygdala	-	-23.0	-4.9	-17.7
	ACC	Cingulate Gyrus anterior division	24,32	-4.5	17.7	24.6
	PCC	Cingulate Gyrus posterior division	23,31	-5.7	-37.8	29.6
	FOC	Frontal Orbital Cortex	10,11,47	-29.5	23.7	-16.6
	HP	Hippocampus	-	-25.2	-23.2	-13.8
	PHa	Parahippocampal Gyrus anterior division	28, 34-36	-21.9	-9.1	-30.3
	SC	Subcallosal Cortex	25	-4.9	20.5	-14.8
	TP	Temporal Pole	38	-40.5	11.1	-29.6

[a] The abbreviations for brain networks: AN = Auditory Network; CEN = Central Executive Network; DAN = Dorsal Attention Network; DMN = Default Mode Network; SN = Salience Network; SMN = Sensorimotor Network; VN = Visual Network; LS = Limbic System.

[b] The abbreviations for ROIs follow the standardized terminology of the National Center for Biotechnology Information (NCBI). Brodmann Areas (BA) and stereotactic coordinates (x, y, z) are provided to facilitate anatomical localization and interpretation of the ROIs. For brevity, we present coordinates only for ROIs in the left hemisphere, except for five midline structures: the Frontal Medial Cortex, Subcallosal Cortex, Precuneus Cortex, anterior division of the Cingulate Gyrus, and posterior division of the Cingulate Gyrus.
